# Removal of tine-based leadless pacemakers: insights from a large multicentre experience

**DOI:** 10.1093/europace/euag157

**Published:** 2026-07-04

**Authors:** Stjepan Jurisic, Jeanne du Fay de Lavallaz, Alessio Gasperetti, Mikhael El-Chami, Marco Schiavone, Ryan Cunnane, Wissam Mekary, Manuel Cerini, Harikrishna Tandri, Antonio Curnis, Martin Jurisic, Gianfranco Mitacchione, Claudio Tondo, Simone Gulletta, Giovanni B Forleo, Giovanni Rovaris, Karim Saleh, Shmaila Saleem-Talib, Hemanth Ramanna, Jean-Claude Deharo, Daniel Hofer, Alexander Breitenstein

**Affiliations:** Department of Cardiology, University Hospital Zurich, Raemistrasse 100, Zurich CH-8091, Switzerland; Division of Cardiac Electrophysiology, University of Michigan Health Care, Ann Arbor, MI, USA; Department of Clinical Electrophysiology & Cardiac Pacing, Centro Cardiologico Monzino, IRCCS, Milan, Italy; Arrhythmology and Electrophysiology Unit, San Raffaele Hospital, IRCCS, Milan, Italy; Division of Cardiology, Section of Electrophysiology, Emory University School of Medicine, 550 W Peachtree St NE, Atlanta, GA 30308, USA; Department of Clinical Electrophysiology & Cardiac Pacing, Centro Cardiologico Monzino, IRCCS, Milan, Italy; Division of Cardiac Electrophysiology, University of Michigan Health Care, Ann Arbor, MI, USA; Division of Cardiology, Section of Electrophysiology, Emory University School of Medicine, 550 W Peachtree St NE, Atlanta, GA 30308, USA; Cardiology Department, Spedali Civili Hospital, University of Brescia, Brescia, Italy; Division of Medicine, Department of Cardiology, Johns Hopkins University, Baltimore, MD, USA; Cardiology Department, Spedali Civili Hospital, University of Brescia, Brescia, Italy; Department of Cardiology, TUM University Hospital German Heart Center, Munich, Germany; Cardiology Department, Spedali Civili Hospital, University of Brescia, Brescia, Italy; Department of Clinical Electrophysiology & Cardiac Pacing, Centro Cardiologico Monzino, IRCCS, Milan, Italy; Department of Biomedical, Surgical and Dental Sciences, University of Milan, Milan, Italy; Arrhythmology and Electrophysiology Unit, San Raffaele Hospital, IRCCS, Milan, Italy; Cardiology Unit, Luigi Sacco University Hospital, Milan, Italy; Cardiology Unit, Fondazione IRCCS San Gerardo dei Tintori, Monza, Italy; Department of Cardiology, Kepler University Hospital Linz, Medical Faculty, Johannes Kepler University Linz, Linz, Austria; Department of Cardiology, Haga Teaching Hospital, The Hague, The Netherlands; Department of Cardiology, Haga Teaching Hospital, The Hague, The Netherlands; Department of Cardiology, CHU Timone, Marseille, France; Department of Cardiology, University Hospital Zurich, Raemistrasse 100, Zurich CH-8091, Switzerland; Department of Cardiology, University Hospital Zurich, Raemistrasse 100, Zurich CH-8091, Switzerland

**Keywords:** Retrieval/Removal, Extraction, Leadless pacemaker

## Abstract

**Aims:**

As implantation rates of leadless pacemakers (LPM) increase, a growing number of patients will require assessment of LPM removability. This multicentre study investigated the safety and feasibility of removal of tine-based LPMs.

**Methods and results:**

We retrospectively analysed consecutive patients who underwent tine-based LPM removal between March 2016 and January 2024 across 11 centres in Europe and the USA. Demographic characteristics, indication for LPM removal, procedural success, and complication rates were assessed. A total of 85 patients [median age 73 years (IQR 64–82)] were included. Indications for LPM removal included temporary use after transvenous lead extraction (23.5%), upgrade to a biventricular pacing system (22.4%) or a transvenous dual-chamber pacemaker (9.4%), and increased pacing thresholds (21.2%). The median device dwell time was 96 days (IQR 29–320 days), and the overall procedural success rate was 96.5%. In the majority of cases, removal was performed using a steerable sheath in combination with a single-loop snare. Procedure-related adverse events occurred in 5.9% of patients, including two pericardial effusions and three cases of device embolization. Significant predictors of removal failure (*n* = 3, 3.5%) were apical device position [odds ratio (OR) 61.8.0; 95% confidence interval (CI) 2.5–788716.3, *P* = 0.008] and longer device dwell time [OR 1.4; 95% CI 1.1–2.4, *P* = 0.008].

**Conclusion:**

Removal of tine-based LPMs, typically after short- to mid-term device dwell times, was generally feasible and safe.

## Graphical Abstract

**Graphical Abstract:**
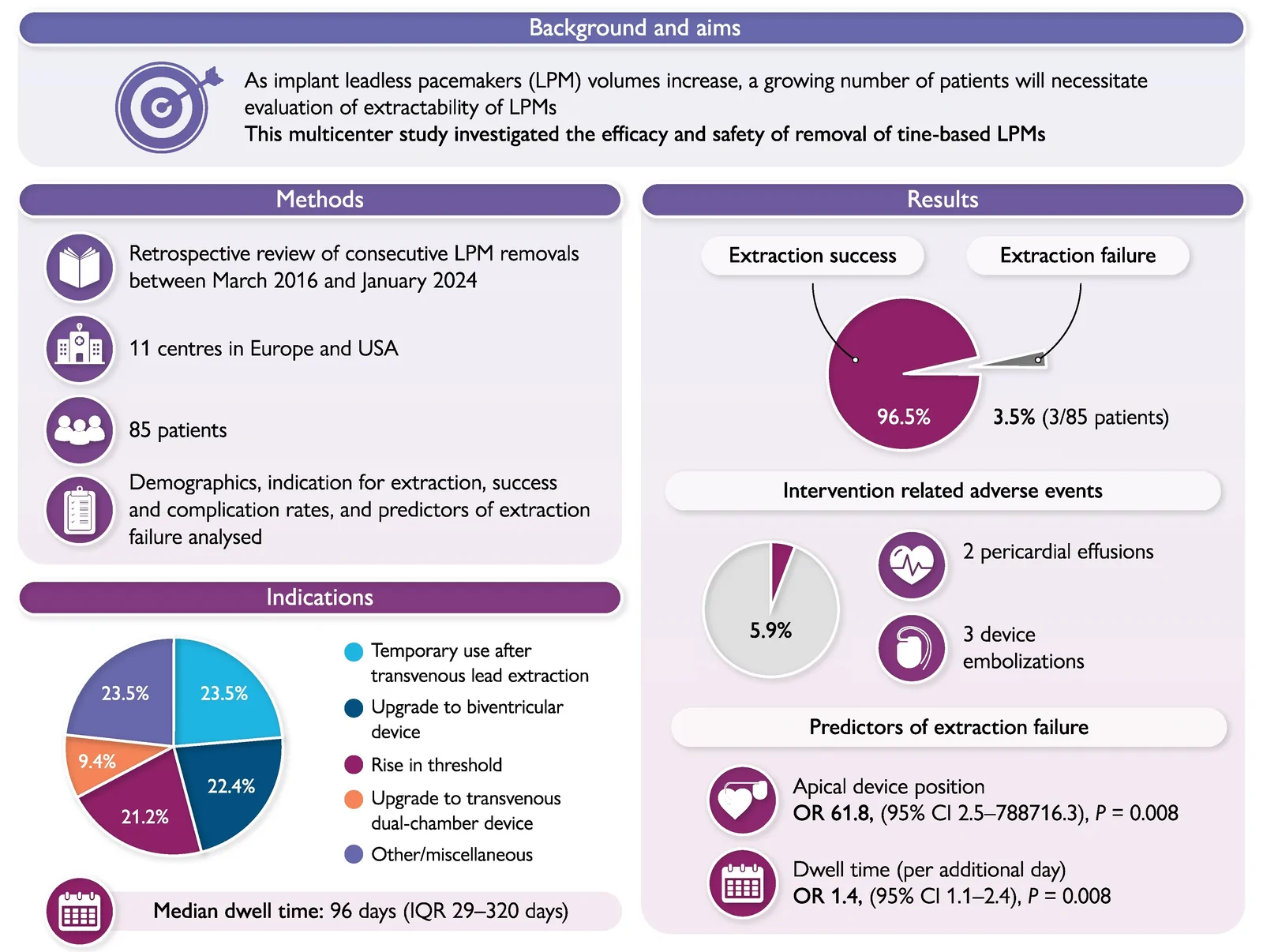

What's New?

- We report the largest multicentre cohort to date evaluating leadless pacemaker removal, demonstrating a procedural success rate of 96.5% after a median device dwell time of 93 days.
- Longer device dwell time and apical position of the leadless pacemakers were independent predictors of removal failure.

## Introduction

Leadless cardiac pacemakers (LPMs) have become an established alternative to conventional transvenous pacemakers (PMs) for selected patients.^1,2^ Studies have demonstrated comparable safety and efficacy of LPMs compared with conventional pacemakers (PM), including in patients undergoing implantation following transvenous pacemaker lead extraction.^3–5^ Two types of leadless pacemakers with different fixation mechanisms (tine- and helix-based) are currently approved for clinical use.^3,6,7^ While malfunctioning or end-of-service LPMs can be deactivated and abandoned in situ, there is a growing need for device removal in selected clinical scenarios. These include patients requiring continued leadless pacing despite limited ventricular size, younger patients in whom long-term device management is of particular importance, the presence of previously implanted intracardiac foreign material such as transvenous PM leads or other LPMs, and, less frequently, suspected device-related infection.^8,9^ Indeed, a recent French cohort analysis including nearly 9000 LPM implantations reported that 1.1% of patients required a revision procedure, with a subset undergoing device removal.^10^ This issue is particularly relevant in younger patients, in whom long-term device management strategies are crucial and the consequences of leaving inactive LPMs in situ remain uncertain.

To date, LPM retrieval has been described mainly in small cohorts and case reports.^11–19^ The largest available study investigating the removability of tine-based LPMs included 18 patients with a median device dwell time of 46 days.^20^ Notably, recent case reports have demonstrated successful extraction of such devices after device dwell times of up to 9 years.^12,15^ More data are available regarding removal of the first-generation helix-based leadless pacemaker systems.^11,19,21^ However, large-scale multicentre data evaluating the feasibility and safety of tine-based LPM removal remain lacking. Therefore, we aimed to analyse a multicentre cohort of patients undergoing tine-based LPM removal to assess the safety and technical success of this procedure and to investigate potential factors associated with removal outcomes.

## Methods

### Terminology

The terms *retrieval* or *explantation* are used for removal of devices with a dwell time < 1 year, whereas *extraction* is the preferred terminology for removal of devices with longer dwell times.^22^ The term *removal* is used as a general heading irrespective of the device dwell time.

### Retrospective patient cohort analysis

**Figure 1:**
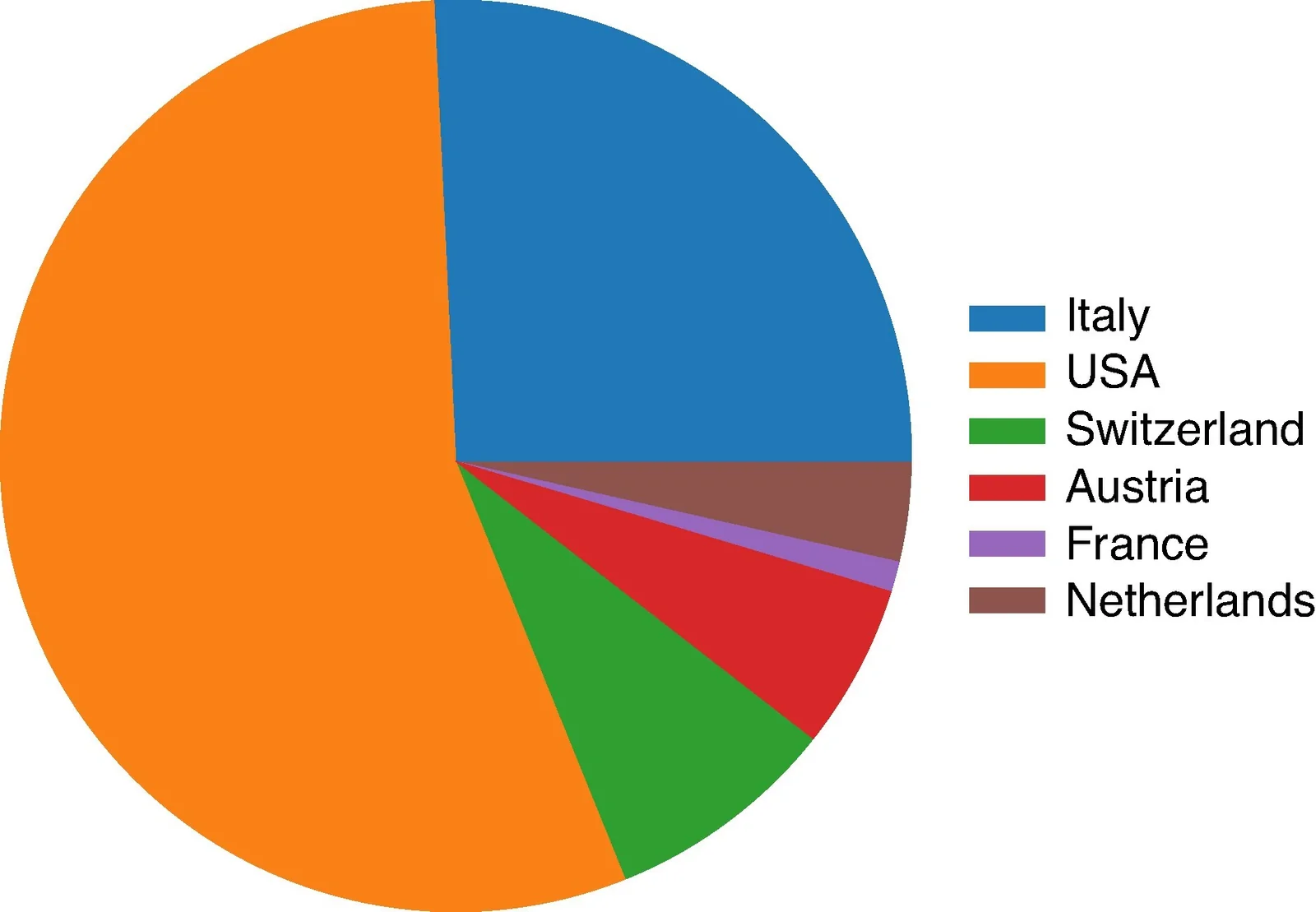
Case distribution across centres.

We retrospectively collected data from consecutive patients who underwent implantation of a tine-based leadless cardiac pacemaker (Micra^TM^ VR or AV, Medtronic Inc., Minneapolis, MN, USA) between March 2016 and January 2024 across 11 centres in Europe and the USA and subsequently underwent device removal (USA, Italy, Switzerland, Austria, the Netherlands, and France; *Figure 1*). The analysis focused on demographic characteristics, indications for LPM removal, procedural success, and complication rates. The study was conducted in accordance with the principles of the Declaration of Helsinki and was approved by the local institutional review boards of all participating centres.

Successful removal was defined as complete explantation/extraction of the targeted device, whereas a failed intervention was defined as a procedure in which the device removal was abandoned and the PM was left in situ.

Continuous variables were summarized as mean (standard deviation) or median [interquartile range (IQR)], as appropriate, while categorical variables were expressed as counts and percentages. Differences between patient groups were assessed using the Student’s *t*-test, Mann–Whitney–Wilcoxon test, or Kruskal–Wallis test for continuous variables and Pearson χ^2^ test for categorical variables. Correlations were analysed using Spearman’s rank correlation coefficient.

Univariable analysis was performed to evaluate patient sex, apical device position, dwell time, and body mass index as clinically relevant variables. Given the low number of outcome events, predictive analyses were additionally calculated using Firth bias-reduced logistic regression, a penalized likelihood method that reduces small-sample bias and provides stable parameter estimates when conventional logistic regression may be unreliable because of sparse data or separation. Candidate variables were selected *a priori* based on their clinical relevance.

All hypothesis tests were two-sided, and a *P*-value <0.05 was considered statistically significant. Statistical analyses were performed using R statistical software (R Foundation for Statistical Computing, Vienna, Austria).

## Procedural technique for leadless pacemaker removal and re-implantation

Device removal was performed via a femoral venous approach. A large bored-sheath (27Fr Micra^TM^ introducer sheath or, in case where the dedicated Abbott AVEIR^TM^ retrieval catheter was used, the 27Fr Abbott AVEIR^TM^ introducer sheath) was used in all cases. Removal was performed using either the Micra™ delivery catheter (when leadless pacemaker re-implantation during the same procedure was planned), a steerable sheath (e.g. Agilis™ NxT, Abbott, Sylmar, CA, USA) in combination with a single- or triple-loop snare (20–25 mm), or the dedicated AVEIR™ retrieval catheter, according to operator preference. When device re-implantation was planned, the new device was generally implanted during the same procedure.

## Results

### Demographic data of patients

Patient, device, and procedural characteristics are summarized in *Table 1*. A total of 85 patients underwent removal of a tine-based LPM after a median device dwell time of 96 days (IQR 29–320 days); Overall, 25.4% of devices had been implanted for >1 year, and 4% for >5 years before removal. Data on the total number of patients evaluated for LPM revision but ultimately not undergoing device removal were not consistently available across participating centres.

**Table 1:** Patient demographic and implantation data

Patient age at the time of LPM removal [years, median (IQR)]	73 (64–82)
Indication for LPM implantation [*n* (%)]	
Complete permanent AV block	52 (61.2)
Intermittent AV block	11 (12.9)
Slow-conducted atrial fibrillation	10 (11.8)
Sick sinus syndrome	5 (5.9)
Tachy-brady syndrome	3 (3.5)
Other indications	4 (4.7)
Device position [*n* (%)]	
Apical RV	17 (20.0)
Inferoseptal RV	11 (12.9)
Midseptal RV	46 (54.1)
Highseptal RV	11 (12.9)
RVOT	0 (0)
Dwell time [days, median, (IQR)]	96 (29–320)
<1 year [*n* (%)]	65 (76.4)
1–2 years [*n* (%)]	8 (9.4)
2–3 years [*n* (%)]	2 (2.4)
3–4 years [*n* (%)]	5 (5.9)
4–5 years [*n* (%)]	2 (2.4)
> 5 years [*n* (%)]	3 (3.5)
Reason for explantation [*n* (%)]	
Temporary implantation (‘bridge-to-decision’)	20 (23.5)
Upgrade to a transvenous biventricular device	19 (22.4)
Elevation in pacing threshold	18 (21.2)
Upgrade to a transvenous dual-chamber device	8 (9.4)
Battery depletion	7 (8.2)
Potential infection	5 (5.9)
Others	8 (9.4)
Retrieval catheter [*n* (%)]	
Steerable sheath	73 (85.9)
Micra^TM^ delivery catheter	7 (8.2)
AVEIR^TM^ retrieval catheter	5 (5.9)
Snare type [*n* (%)]	
Goose Neck^TM^	40 (47.1)
EN Snare®	29 (34.1)
ONE Snare®	6 (7.1)
Other	10 (11.8)
Success rate [*n* (%)]	
Successful removal	82 (96.5)
Non-successful removal	3 (3.5)

The median patient age at the time of removal was 73 years (IQR 64–82 years). Coronary artery disease was present in 38% of patients, while 18% suffered from dilated cardiomyopathy. The main indications for LPM implantation were permanent complete atrioventricular (AV) block (61.2%, *n* = 52), intermittent complete AV block (12.9%, *n*=11), slow-conducted atrial fibrillation (11.8%, n=10), sick sinus syndrome (5.9%, n=5), and tachycardia-bradycardia syndrome (3.5%, n=3). Device location was the right ventricular (RV) apex in 17 patients (20.0%), the inferoseptal region in 11 (12.9%), the mid-septum in 46 (54.1%), and the high septum in 11 (12.9%).

### Reason for LPM removal

Clinical scenarios prompting Micra^TM^ retrieval were categorized as infection-related, upgrade-related, or other indications. The principal rationale for device removal was to minimize the long-term burden of intracardiac foreign material within the RV. Infection-related scenarios included temporary leadless pacemaker implantation following extraction of an infected transvenous CIED (‘bridge-to-decision’) in 20 patients (23.5%). A clinical need for device upgrade was present in 27 patients (31.8%), while suspected leadless device infection was the indication for removal in five patients (5.9%). Other indications included elevated pacing thresholds (21.2%) and battery depletion (8.2%), with removal performed to avoid retention of unnecessary intracardiac foreign material.

### Procedural success and complications

The overall procedural success rate was 96.5% (82/85 patients; *Table 1*). General anaesthesia was used in 23 patients (27.1%), whereas the remaining procedures were performed under operator-guided sedation. Device removal was performed using a steerable sheath in 73 cases (85.9%), the Micra^TM^ delivery catheter in seven (8.2%), and the AVEIR^TM^ retrieval catheter in five (5.9%). Snare systems included a single-loop Goose Neck^TM^ snare in 40 procedures (47.1%), a triple-loop EN Snare® in 29 (34.1%), and a ONE Snare® in six (7.1%). Immediate device re-implantation was performed in 79 patients (92.9%), of whom 34.2% received a new LPM. Mean procedural duration was 125 ± 66 minutes, including procedures in which device reimplantation was performed.

Device removal was unsuccessful in three patients (3.5%). In two cases, the proximal retrieval feature could not be engaged despite repeated attempts using a steerable sheath with 20-mm and 25-mm snares. These devices had relatively short dwell times (50 and 44 days), and retrieval failure was considered most likely due to unfavourable device orientation and/or entrapment within the right ventricular trabeculations, preventing secure snare engagement. In the third case, the device had been implanted for 3140 days. Although the proximal retrieval feature was successfully captured, extensive fibrotic encapsulation prevented extraction despite multiple extraction attempts (*Table 2*). In all three cases, the procedures were terminated and the devices were left in situ.

**Table 2:** Patient-level procedural complications during leadless pacemaker removal (ID #1–5) and unsuccessful removals (ID #6–8)

ID	Age [years]	Dwell time [days]	Device position	Complication type/ Reason for unsuccessful device removal	Management	Clinical Outcome
#1	74	127	Apical	Pericardial effusion	Pericardial drainage	Recovered
#2	80	1477	High-septal	Pericardial effusion	Conservatively (no intervention)	Recovered
#3	90	1449	Apical	Device embolization to coronary sinus	Surgical retrieval	Recovered
#4	87	1	Infero-septal	Device embolization to pulmonary circulation	Snared from pulmonary artery via pre-existing introducer sheath	Successful
#5	77	42	Apical	Device embolization to pulmonary circulation	Snared from pulmonary artery via second access from the left femoral vein	Successful
#6	85	3140	Mid-septal	Extensive fibrotic adhesions, tines probably subperforated into pericardial space	Left in situ	Not successful
#7	85	44	Apical	Proximal retrieval feature could not be engaged	Left in situ	Not successful
#8	67	50	Apical	Proximal retrieval feature seemed buried in trabeculae	Left in situ	Not successful

The overall complication rate was low, with two pericardial effusions, one of which required pericardial drainage (*Table 2*). Device embolization occurred in three procedures owing to loss of device control during retrieval. In two cases, the device was immediately recaptured percutaneously without clinical sequelae. In the remaining case, the device embolized into the coronary sinus and required surgical removal.

In univariable analysis, only device dwell time was significantly associated with removal failure (see Supplementary Material online, *Tables S1*  *and S2*). The additionally performed Firth biased-reduced logistic regression, a statistical method mitigating biases rooted in small sample sizes, assessing the predictive value of BMI, patient sex, dwell time, and position of the device (apical vs. non-apical) demonstrated that apical device position [odds ratio (OR) 61.8; 95% confidence interval (CI) 2.5–788716.3, *P* = 0.008; *Table 3*] and longer device dwell time (OR 1.4 per 180 days increase; 95% CI 1.1–2.4, *P* = 0.008) were independently associated with removal failure. For both analysis it has to be mentioned that the low number of failed removals limits statistical analyses and these results need to be interpreted with caution.

**Table 3:** Firth logistic regression assessing the predictive value for unsuccessful LPM removal

Variable	OR (95% CI)	*P* value
(Intercept)	0 (0, 0.2)	0.020
Male sex	1.6 (0.1, 25.2)	0.71
Dwell time	1.4 (1.1, 2.4)	0.008
Apical device position	61.8 (2.5, 788716.3)	0.008
BMI	1.2 (0.9, 1.6)	0.28

## Discussion

To our knowledge, this is the largest multicentre study to date evaluating removal of tine-based LPMs across centres in Europe and the USA. Our findings provide important insights into the safety, feasibility, and technical aspects of LPM removal in contemporary clinical practice.

The main findings of this study are as follows:

1. LPM removal was associated with a high procedural success rate of 96.5% after a median device dwell time of 93 days;
2. longer device dwell time and an apical implantation site were independently associated with removal failure; and
3. the most frequent indications for LPM removal were device upgrade and removal of temporarily implanted devices used as a bridge strategy following extraction of infected transvenous CIEDs.

## Clinical indications for LPM removal and factors influencing procedural feasibility

LPM technology has substantially expanded the treatment options for patients with bradyarrhythmias,^23–26^ and implantation rates continue to increase.^27^ Despite the favourable safety and performance profile of current-generation devices, the optimal management of LPMs at end of service (EOS) remains uncertain.^28^ Although device abandonment is currently generally recommended, removal may be considered in selected clinical scenarios, including device infection, device-related complications, or in younger patients in whom the cumulative burden of intracardiac hardware is of particular concern.^1,29,30^ Consistent with this concept, recent registry data demonstrated that approximately 1.1% of patients underwent device revision following Micra^TM^ implantation, with device removal required in a subset of these cases.^10^ Reported indications for LPM removal include device dislocation or embolization,^31^ suspected device infection,^18^ life-threatening device-related complications (e.g. ventricular arrhythmias^32,33^ or right-sided heart failure secondary to RV outflow tract obstruction^13^), and clinical situations in which minimization of intracardiac hardware is desirable, particularly in younger patients.^34^ An emerging indication for LPM implantation is permanent or temporary pacing following extraction of infected transvenous CIED systems. In the present cohort, temporary LPM implantation as a bridge strategy after transvenous CIED extraction represented one of the most common indications for subsequent device removal. Recent studies have demonstrated the feasibility and safety of concomitant leadless pacemaker implantation during extraction of infected transvenous CIEDs (‘two-in-one’ strategy), particularly in pacemaker-dependent patients.^35,36^ Another frequent indication for LPM removal was device upgrade to a biventricular or dual-chamber transvenous pacing system. Removal in this setting was primarily performed to avoid leaving an inactive leadless device within the RV after implantation of a transvenous system. This strategy is consistent with the recent EHRA consensus statement, which emphasizes the importance of longitudinal follow-up after LPM implantation to identify patients who may require device upgrade because of disease progression or evolving pacing requirements.^37^

## Factors influencing leadless pacemaker removal

The feasibility of LPM removal is largely determined by the degree of fibrotic encapsulation and the strength of anchoring at the tine fixation sites. Case series and post-mortem studies confirm a fibrotic response to the device surface,^14,28,38–43^ ranging from minimal tissue adhesion to complete encapsulation.^28^ While some devices become extensively encapsulated within the first year after implantation, others remain only minimally adherent despite substantially longer dwell times. The extent of encapsulation is likely influenced by implantation-, device-, and patient-related factors.

### Implantation-related factors

**Figure 2:**
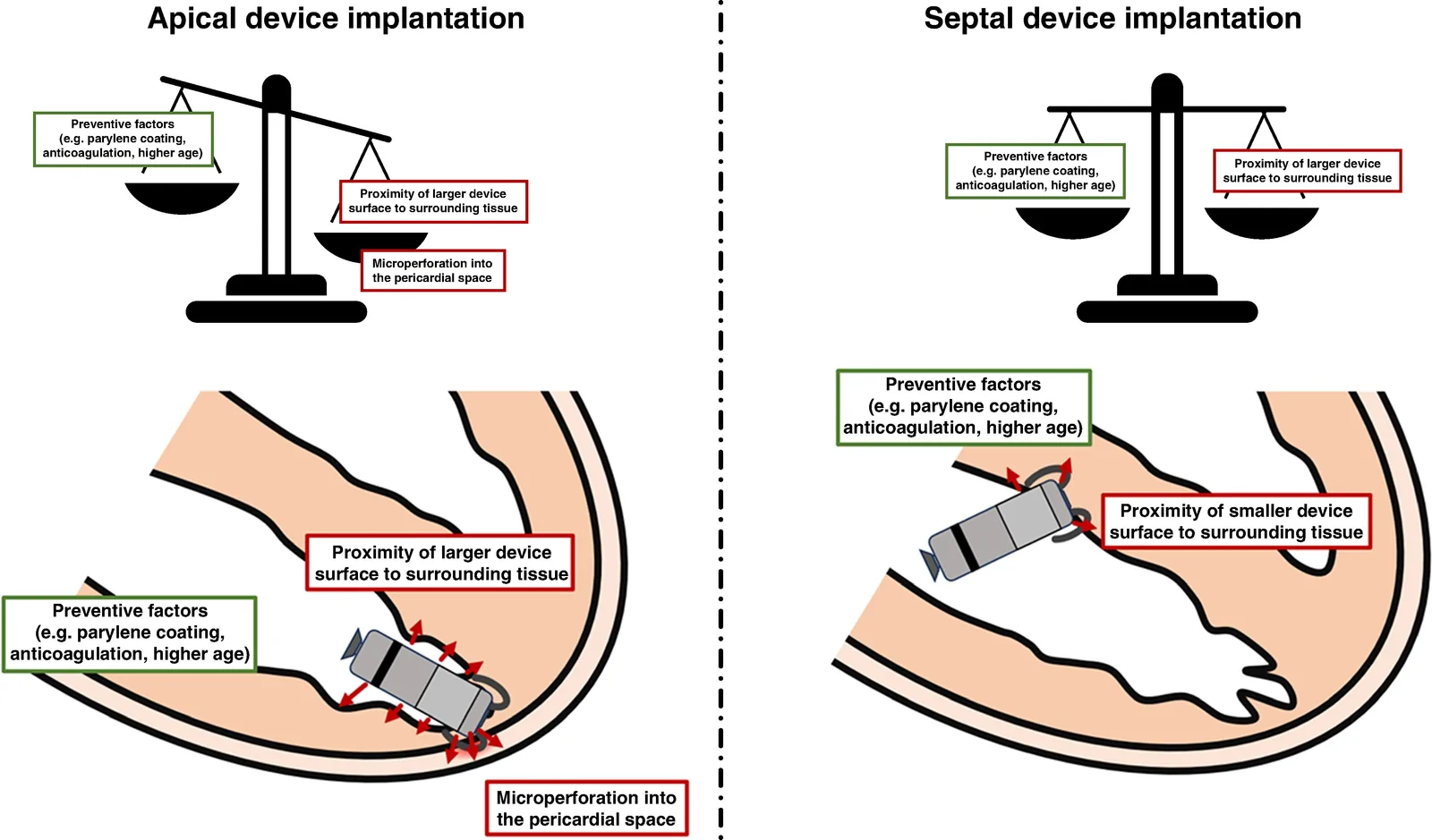
Factors influencing the formation of device surface adhesive tissue: implantation site, device-related, and patient-related factors.

The anatomical site of implantation may substantially influence device removability. The RV apex is characterized by dense trabeculation, whereas the interventricular septum provides a comparatively smoother endocardial surface.^44^ Accordingly, apically implanted devices may become more deeply embedded within the trabecular myocardium, resulting in greater contact between the device and the endothelium (*Figure 2*). Continuous cardiac motion generates chronic mechanical stress at the device-tissue interface, prompting endothelial injury, inflammation, thrombus formation, and subsequent fibrotic encapsulation.^28,45–47^ This mechanism may be more pronounced in apically implanted devices than in septally implantated devices, where a larger proportion of the device body remains relatively ‘free-floating’. In addition, the RV apex is characterized by a thinner myocardial wall, which may increase the risk of myocardial perforation and cardiac tamponade during implantation.^44^ Partial tine penetration beyond the myocardium may remain clinically silent in the absence of electrical abnormalities and significant pericardial effusion *(Figure 2)*.^48^ Although the incidence of these micro-perforations is unknown, they are likely under-recognized. Such injuries may trigger a localized inflammatory response, including post-cardiac injury syndrome,^49^ resulting in fibrosis and firm tissue anchoring that may impair subsequent device removal, even in the absence of extensive encapsulation of the device body. Notably, septal implantation, particularly at more basal regions, may also present challenges for removal. In a previous study evaluating a helix-based leadless pacing system, apically implanted devices demonstrated greater fluoroscopic mobility (‘swinging), suggesting less tissue adherence than mid-septal implants.^19^ This observation appears to contrast with the mechanisms proposed for tine-based devices and may reflect differences in fixation mechanism and device design. Alternatively, basal septal devices may become adherent to or entrapped within the subvalvular apparatus of the tricuspid valve, thereby limiting device mobility and complicating removal. However, direct comparisons between fixation systems remain limited, and the influence of implantation site on long-term removability requires further investigation.

### Device-related factors

Device material and surface characteristics may influence the degree of fibrotic encapsulation. The Micra™ device is coated with parylene C, a biocompatible polymer that may attenuate the local inflammatory response and thereby reduce fibrotic tissue formation.^46,47^ In addition to its favourable biocompatibility, parylene has been shown to reduce bacterial adhesion to implanted medical devices, which may further improve long-term tissue compatibility.^50^ Evidence from transvenous lead extraction also supports the importance of device surface characteristics. For example, implantable cardioverter-defibrillator leads incorporating expanded polytetrafluoroethylene (ePTFE)-coated coils demonstrate reduced fibrotic binding, facilitating lead extraction compared with conventional metallic coils.^51^ These observations suggest that minimizing direct contact between metallic device components and the surrounding tissue may reduce fibrotic encapsulation and improve long-term removability.

### Patient-related factors

Individual predisposition to scar formation is likely to influence device encapsulation. As this process is driven by inflammation, a more pronounced inflammatory response may result in increased fibrotic tissue formation. This has been well documented in paediatric transvenous lead extraction, where more extensive fibrotic adhesions are typically observed,^52–57^ and may similarly affect younger patients with leadless pacemakers. Local thrombus formation following mechanical trauma represents another important contributor to encapsulation.^47^ Conversely, chronic oral anticoagulation has been associated with reduced fibrotic encapsulation and less venous narrowing in patients undergoing transvenous lead extraction, suggesting that it may also influence encapsulation of leadless pacemakers.^58^

## Procedural aspects of LPM removal

**Figure 3:**
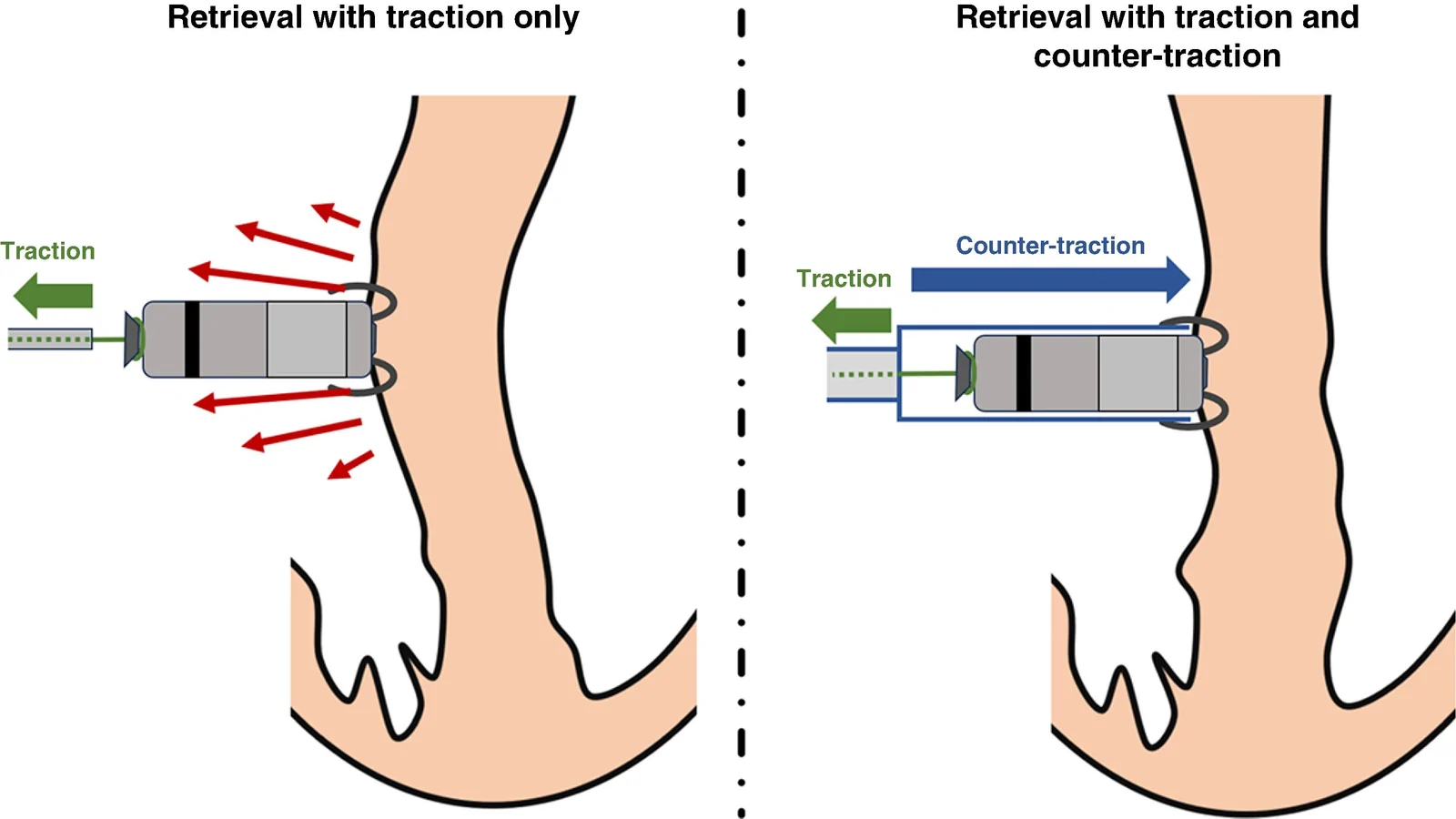
Schematic overview of device removal using either a steerable sheath (left) or the Micra^TM^ delivery catheter (right).

**Figure 4:**
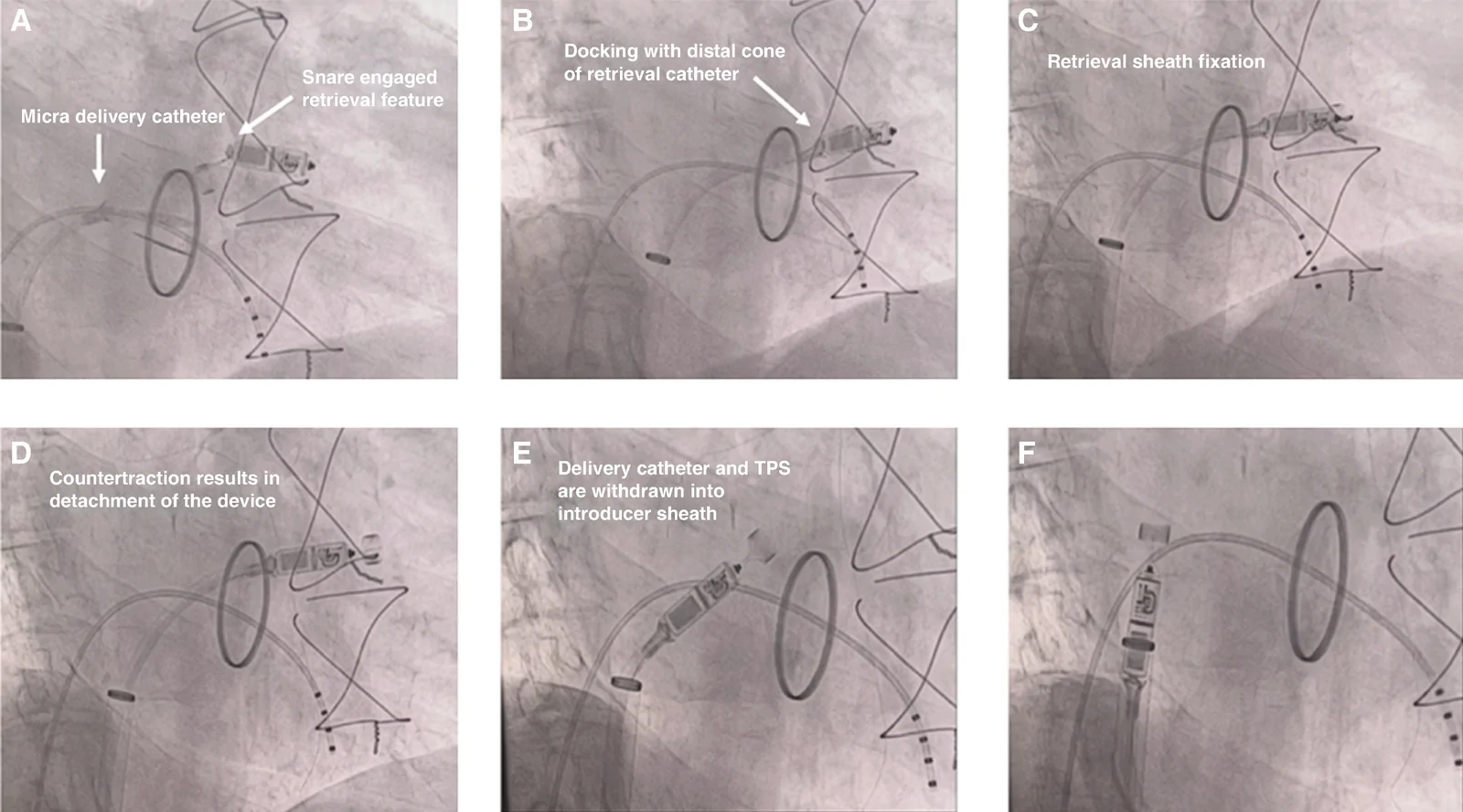
Device removal using the Micra^TM^ delivery catheter in a patient with previous mechanical mitral valve replacement.

Various removal techniques were used in this cohort, with the choice of approach depending on device availability and whether re-implantation was planned. When re-implantation is intended, the Micra^TM^ delivery catheter is frequently used for device removal.^17,59,60^ In this approach, the new device is implanted first.^61^ After deployment, a small (7 mm) single- or triple-loop snare is advanced through the delivery catheter to engage the retrieval feature of the existing device, while the delivery cup provides countertraction and encloses the device during withdrawal, potentially reducing interaction with the tricuspid valve apparatus *(Figures 3* and *4).* However, this approach is limited by the restricted steerability and relative rigidity of the delivery system.

**Figure 5:**
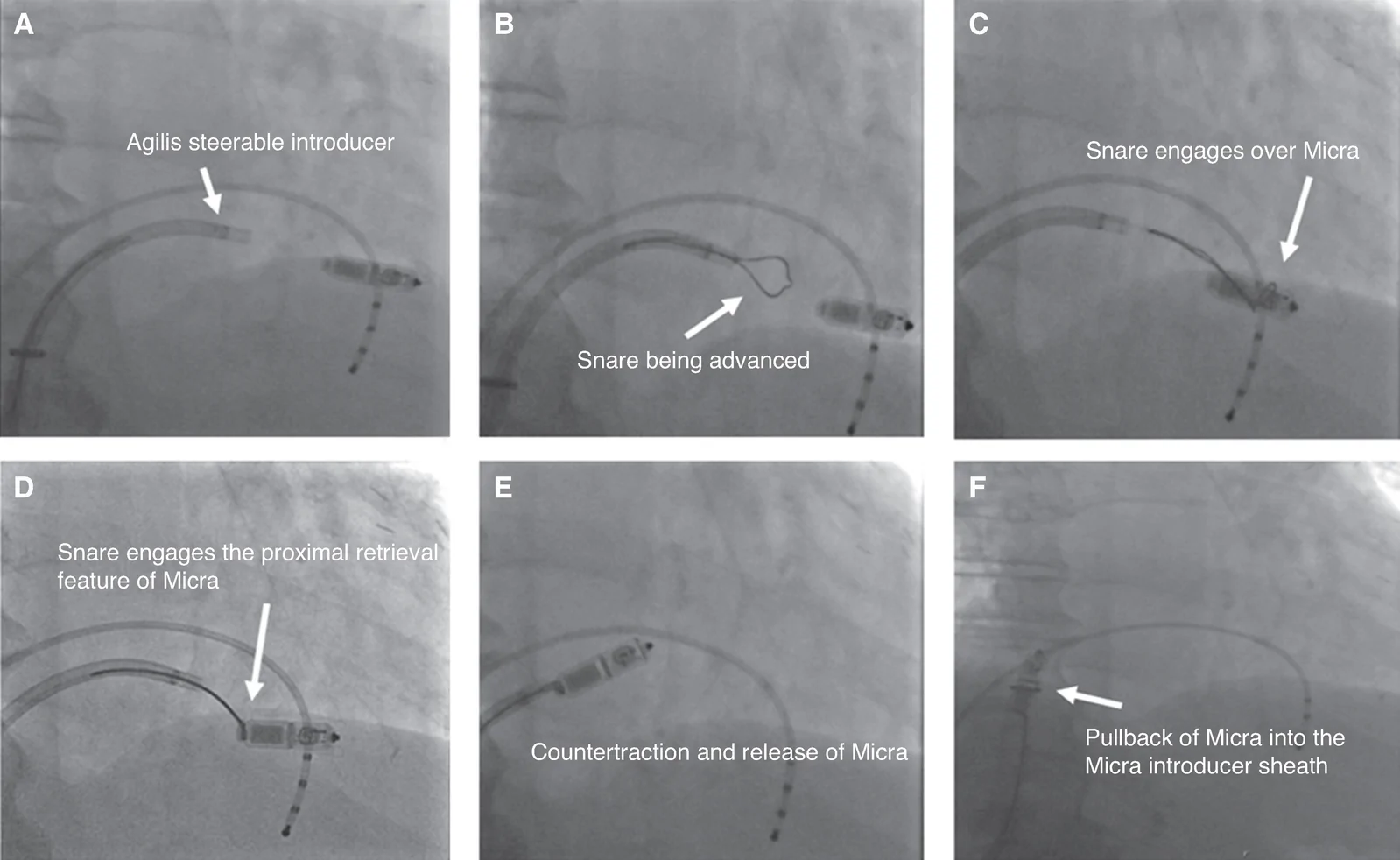
Device removal using a steerable sheath and a GooseNeck^TM^ snare.

When implantation of a new Micra™ was not planned, retrieval was typically performed using a steerable sheath (e.g. Agilis^TM^ NxT, Abbott) in combination with a single- or triple-loop snare^14,59,60^ (*Figures 3* and *5*).^62^ This approach provides greater manoeuvrability and permits the use of larger snare sizes but does not provide countertraction and leaves the fixation tines exposed during removal, potentially increasing interaction with the tricuspid valve apparatus (*Figures 3* and *5*).^63^ This may be clinically relevant, as experience from transvenous lead extraction has shown that mechanical interaction with the tricuspid valve can result in new or worsening tricuspid regurgitation.^64,65^

A third approach involves the dedicated retrieval catheter developed for the helix-based AVEIR^TM^ system^11,66,67^. Following successful device capture, the catheter advances a protective sheath over the device, thereby providing a degree of countertraction while enclosing the fixation mechanism during removal.

Countertraction is a fundamental principle of transvenous lead extraction^68^ and may similarly improve procedural success and safety during leadless pacemaker removal, particularly in devices with longer dwell times. Although prospective comparative data are lacking, dedicated retrieval systems may therefore offer advantages in selected clinical secenarios.

## Safety and efficacy of LPM removal

This analysis demonstrates that short- to mid-term removal of tine-based leadless pacemakers is generally feasible and safe, with an overall procedural success rate of 96.5% after a median device dwell time of 93 days. These findings are consistent with previous studies reporting high removal success rates for both helix- and tine-based leadless pacing systems.^11,21,13–16,69,70^ In our cohort, longer device dwell time and apical implantation were associated with removal failure, likely reflecting more advanced fibrotic encapsulation and stronger device anchoring.^19^ Because longer dwell times frequently coincided with apical implantation, the individual contribution of each factor remains difficult to distinguish. Overall, leadless pacemaker removal appears to be a safe procedure within the range of implantation durations evaluated in this study, with low complication rates consistent with previous reports.^11,13–16,19,21,69,70^ Importantly, the reported success rate reflects technical success in patients in whom removal was attempted and should not be interpreted as the overall success rate of leadless pacemaker revision strategies.

Procedure-related adverse events were infrequent but clinically relevant, occurring in 5.9% of patients. Complications included two pericardial effusions, one of which required percutaneous drainage, as well as three device embolizations, including one case requiring surgical retrieval. These findings emphasize that, despite the high procedural success rate, leadless pacemaker removal should be performed in experienced centres with immediate access to advanced percutaneous treatment options and cardiac surgical backup.

## Limitations

Several limitations should be acknowledged. First, this was a retrospective analysis based on data collected during routine clinical practice, and some patient records were incomplete. Second, as the device dwell times in this study were relatively short, the generalizability of our findings to devices with substantially longer dwell times is limited. Third, the very low number of removal failures substantially constrained the statistical analyses and limited the strength of inferences regarding predictors of removal failure. Most importantly, this study focused on the technical feasibility and safety of retrieving tine-based leadless pacemakers. It was not designed to inform end-of-service management strategies, as only patients who ultimately underwent a removal attempt were included, whereas potential candidates who did not proceed to removal were not captured.

## Conclusion

To our knowledge, this study represents the largest multicentre analysis of tine-based LPM removal to date. Removal of tine-based LPM after short- to mid-term implantation duration is a safe, feasible, and effective procedure with high procedural success and low complication rates.

## Supplementary Material

euag157_Supplementary_Data

## Data Availability

Data are available upon reasonable request made to the corresponding author.
