# Supplementary material for: Removal of tine-based leadless pacemakers: insights from a large multicentre experience
Source: Europace. 2026 Jul 4;28(8):euag157. doi: 10.1093/europace/euag157 (PMC13447091; doi:10.1093/europace/euag157)
Supplement: Supplementary file 1 [file euag157_supplementary_data.zip › Supplementary tables.docx]

# Supplementary tables.

# Supplementary table 1. Failed extraction

| **Characteristic** | **N** | **No**, N = 82^1^ | **Yes**, N = 3^1^ | **p-value**^2^ |
| --- | --- | --- | --- | --- |
| Sex | 85 |  |  | 0.5 |
| Woman |  | 29 / 82 (35%) | 0 / 3 (0%) |  |
| Man |  | 53 / 82 (65%) | 3 / 3 (100%) |  |
| Apical position | 85 | 15 / 82 (18%) | 2 / 3 (67%) | 0.10 |
| BMI | 85 | 26.09 (24.11, 29.65) | 30.60 (27.98, 30.62) | 0.3 |
| Dwell time in months | 85 |  |  | 0.078 |
| 0-6 months |  | 53 / 82 (65%) | 1 / 3 (33%) |  |
| 7-12 months |  | 11 / 82 (13%) | 0 / 3 (0%) |  |
| 13-18 months |  | 5 / 82 (6.1%) | 0 / 3 (0%) |  |
| 19-24 months |  | 3 / 82 (3.7%) | 0 / 3 (0%) |  |
| 25-30 months |  | 1 / 82 (1.2%) | 0 / 3 (0%) |  |
| 31-36 months |  | 1 / 82 (1.2%) | 0 / 3 (0%) |  |
| 37-42 months |  | 2 / 82 (2.4%) | 0 / 3 (0%) |  |
| 43-48 months |  | 1 / 82 (1.2%) | 0 / 3 (0%) |  |
| 49-54 months |  | 3 / 82 (3.7%) | 1 / 3 (33%) |  |
| 73-78 months |  | 1 / 82 (1.2%) | 0 / 3 (0%) |  |
| 79-84 months |  | 1 / 82 (1.2%) | 0 / 3 (0%) |  |
| 103-108 months |  | 0 / 82 (0%) | 1 / 3 (33%) |  |
| ^1^Median (p25, p75), mean (sd) or Frequency (%) | | | | |
| ^2^Fisher's exact test; Wilcoxon rank sum test | | | | |

# Supplementary table 2. Bivariable analysis

| Variable | Estimate, CI | p-value |
| --- | --- | --- |
| Male sex | 48369929.961, 95%CI [0, NA] | 0.996 |
| Dwell time, per 180d increase | 1.347, 95%CI [1.085, 1.759] | 0.009 |
| apical position | 8.933, 95%CI [0.806, 199.812] | 0.082 |
| BMI | 1.058, 95%CI [0.84, 1.252] | 0.555 |

# Supplementary table 3. Table according to dwell time

| **Characteristic** | **N** | **less than 1 year**, N = 65^1^ | **more or equal 1 year**, N = 20^1^ | **p-value**^2^ |
| --- | --- | --- | --- | --- |
| Type of retrieval catheter | 85 |  |  | 0.2 |
| Steerable sheath (e.g. Agilis) |  | 58 / 65 (89%) | 15 / 20 (75%) |  |
| Micra delivery catheter |  | 4 / 65 (6.2%) | 3 / 20 (15%) |  |
| AVEIR |  | 3 / 65 (4.6%) | 2 / 20 (10%) |  |
| Type of snare | 85 |  |  | 0.033 |
| EnSnare |  | 27 / 65 (42%) | 2 / 20 (10%) |  |
| GooseNeck Snare |  | 28 / 65 (43%) | 12 / 20 (60%) |  |
| OneSnare |  | 4 / 65 (6.2%) | 2 / 20 (10%) |  |
| Others |  | 6 / 65 (9.2%) | 4 / 20 (20%) |  |
| Successful explant | 85 | 64 / 65 (98%) | 18 / 20 (90%) | 0.14 |
| Complication | 85 |  |  | 0.6 |
| No |  | 61 / 65 (94%) | 18 / 20 (90%) |  |
| Pericardial effusion |  | 1 / 65 (1.5%) | 1 / 20 (5.0%) |  |
| Tricuspid valve damage |  | 0 / 65 (0%) | 0 / 20 (0%) |  |
| Embolisation of explanted device |  | 3 / 65 (4.6%) | 1 / 20 (5.0%) |  |
| Others |  | 0 / 65 (0%) | 0 / 20 (0%) |  |
| Failed extraction | 85 | 1 / 65 (1.5%) | 2 / 20 (10%) | 0.14 |
| ^1^Median (p25, p75), mean (sd) or Frequency (%) | | | | |
| ^2^Wilcoxon rank sum test; Pearson's Chi-squared test; Fisher's exact test | | | | |
